# The Effects of Bile Duct Obstruction on Liver Volume: An Experimental Study

**DOI:** 10.1155/2013/156347

**Published:** 2013-06-05

**Authors:** Bahtiyar Ertor, Serdar Topaloglu, Adnan Calik, Umit Cobanoglu, Ali Ahmetoglu, Huseyin Ak, Erdem Karabulut, Mithat Kerim Arslan

**Affiliations:** ^1^Department of Surgery, Karadeniz Technical University, School of Medicine, 61080 Trabzon, Turkey; ^2^Department of Pathology, Karadeniz Technical University, School of Medicine, 61080 Trabzon, Turkey; ^3^Department of Radiology, Karadeniz Technical University, School of Medicine, 61080 Trabzon, Turkey; ^4^Department of Biostatistics, Hacettepe University, School of Medicine, 06100 Ankara, Turkey

## Abstract

*Objectives.* This study is aimed at investigating alterations in liver volume during obstructive jaundice in rat liver. *Materials and Methods.* Thirty-six rats were divided into four groups. Abdominal tomography was performed for baseline volumetric analyses. The main bile ducts were ligated (BDL). Volumetric analyses were repeated 3 days after BDL in group 1, 7 days after BDL in group 2, 15 days after BDL in group 3, and 25 days after BDL in group 4, and total hepatectomy was performed in all animals. Control group (*n* = 4) was created with the rats that died before bile duct ligation. *Results.* There was no difference found in liver volume in group 1 compared to control animals. The liver volume was increased 7 days after BDL (*P* = 0.01). It was increased up to 60% of baseline values 25 days after BDL (*P* = 0.002). Wet liver weights of animals were also increased compared to control group. Liver weights were increased up to 40% percent of baseline values in group 4 (*P* = 0.002). *Conclusions.* Liver volume and weight were increased after BDL. Liver surgery in patients with huge liver mass is generally associated with significant difficulty. The surgeon should be aware of the time-dependent alteration in liver volume after obstructive jaundice.

## 1. Introduction

Chronic cholestatic liver disease and bile duct tumors are the main causes of chronic cholestasis in daily clinical practice. Postcanalicular biliary obstruction leads to bile duct epithelial cell proliferation and periportal fibrosis [1–3]. Clinical and experimental studies have found that only the timely restoration of bile flow can halt fibrosis and reverse biliary hyperplasia [4–6]. The role of bile duct obstruction on liver volume has not been evaluated in detail. Thus, the aim of this study was to identify the time-dependent alterations on liver volume after bile duct ligation.

## 2. Materials and Methods

Thirty-six male Sprague-Dawley rats weighing between 180 and 300 g were used for the study. They were kept under routine laboratory conditions and received standard laboratory chow with free access to food and water. The study protocol was approved by the institutional Ethics Committee for Experimental Studies at February 3, 2010, document number: 01/2010.

### 2.1. Experimental Design

The animals were divided into four groups of 8 animals. Weight measurement and abdominal tomography under intraperitoneal ketamine (50 mg/kg, Ketalar, Parke-Davis, Ann Arbor, Michigan, USA) anesthesia for volumetric analyses were performed before bile duct ligation (BDL). In group 1 (*n* = 8), abdominal tomography (CT) was performed 3 days after BDL for reevaluation of volumetric status. Weight of animals was remeasured, and total hepatectomy was performed for determination of wet liver weight. In group 2 (*n* = 8), abdominal CT was performed 7 days after BDL. Weight of animals was remeasured and total hepatectomy was performed. In group 3 (*n* = 8) abdominal CT was performed 15 days after BDL. During the followup period, animals received subcutaneous injections of vitamin K1 (50 g) at 8th according to suggestions of Beck and Lee [7]. Weight of animals was remeasured, and total hepatectomy was performed. In group 4 (*n* = 8), abdominal CT was performed 25 days after BDL. During the follow-up period, animals received subcutaneous injections of vitamin K1 (50 g) at 8th, 16th, and 21st days after BDL. Four animals died after administration of ketamine before BDL. These were considered as a control group. The experiment was repeated for dead animals. Histopathological analysis of liver was also performed in all groups. 

### 2.2. Surgical Procedure

Animals were fasted for 12 hours before the experiments; however, they were allowed to drink water. All surgical procedures were performed under 50 mg/kg ketamine anesthesia. A midline abdominal incision was preferred for laparotomy. Portal pedicle dissection was performed. Common bile duct was ligated with 3/0 silk suture. Laparotomy incision and skin incision were separately closed with 2/0 silk sutures. Animals were followed in separate follow-up cages. The same incision was used for relaparotomy and total hepatectomy.

### 2.3. Volumetric Analyses

All CT examinations were performed on a 16-slice CT system (Somatom Sensation, Siemens, Erlangen, Germany). The scan protocol was as follows: slice collimation, 16 × 1.5 mm; table feed/rotation, 18.0 mm; rotation time, 0.5 s; 120 Kv; effective 200 mAs. Images were reconstructed with a slice thickness of 1 mm in axial plane. Volumetric analyses were performed with special software program for CT (Syngo Volume Evaluation version B10/2004A, Siemens Medical, Germany). Volumetry of the liver on CT images was performed by manual tracing of the liver boundary and summation of the liver area on each section by an experienced radiologist on liver imaging.

### 2.4. Histological Procedures

Fresh livers of animals were fixed with 10% formaldehyde. The fixed tissue embedded in paraffin was processed for light microscopy. Sections were obtained from each of the three pieces of liver to evaluate histopathologic damage. Sections of 5 *μ*m were deparaffinized and dyed with hematoxylin-eosin for structural examination of liver. The same sections were also stained with Masson's trichrome for evaluation of fibrosis. All examinations were performed under a light microscope (Zeiss, Gottingen, Germany). 

### 2.5. Statistical Analyses

All values are expressed as mean ± SEM (standard error of mean). The one-way ANOVA test was used for basic comparison of data. If differences were found to be significant, paired *t*-test was used for further analyses. *P* values less than 0.05 were considered significant. 

## 3. Results

**Figure 1:**
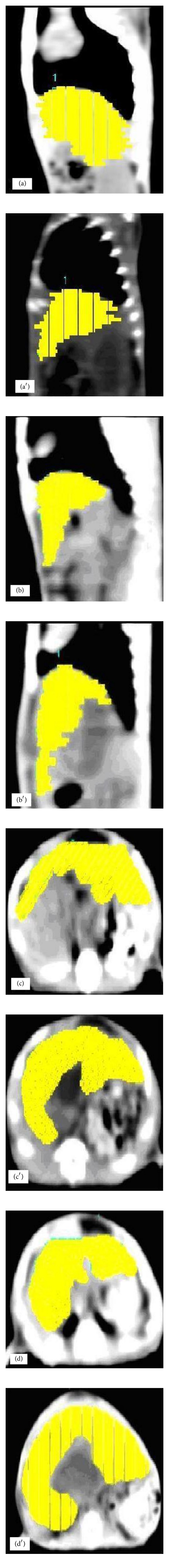
Photos of volumetric analysis performed by CT scan according to experimental groups. Most demonstrative samples were selected from each group. (a) Volumetric analysis of liver before BDL (7.45 cm^3^, 2nd animal in group 1). (a′) Volumetric analysis of liver 3 days after BDL (7.97 cm^3^, 2nd animal in group 1). (b) Volumetric analysis of liver before BDL (4.14 cm^3^, 4th animal in group 2). (b′) Volumetric analysis of liver 7 days after BDL (5.77 cm^3^, 4th animal in group 2). (c) Volumetric analysis of liver before BDL (5.06 cm^3^, 8th animal in group 3). (c′) Volumetric analysis of liver 15 days after BDL (7.56 cm^3^, 8th animal in group 3). (d) Volumetric analysis of liver before BDL (5.50 cm^3^, 6th animal in group 4). (d′) Volumetric analysis of liver 25 days after BDL (12.16 cm^3^, 6th animal in group 4).

**Figure 2:**
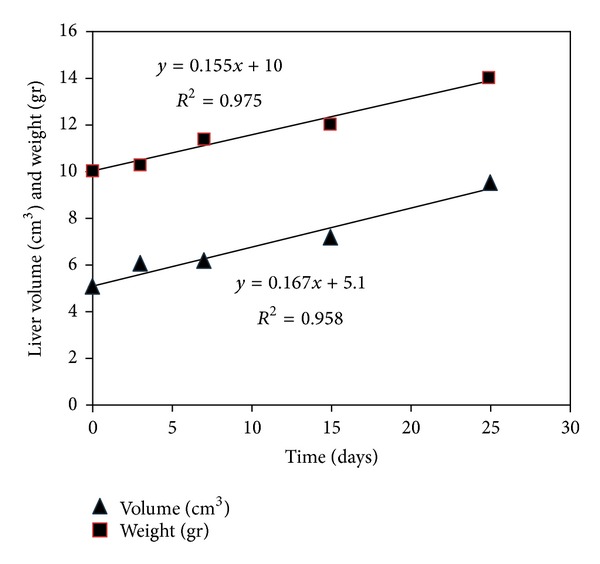
Time dependent alteration of liver volumes and weights after BDL.

Before the experiment, homogenous distribution of animal weights was observed in each group (*P* > 0.05). Experimental protocol did not cause alteration in the weight of animals (Table 1). All animals that underwent BDL were survived until the completion of experiment. Volumetric diversity before and after BDL was determined during 7 days after BDL as an insignificant. However, the difference was became significant at 15 and 25 days after BDL (Table 2) (Figure 1). To refrain from unnecessary sacrifice, the 4 animals that died during anesthetic induction were considered as a control group. The mean weight of animals in control group was 200 gr (±15) (*P* > 0.05, when compared to other experimental groups). The wet liver weight was increased after BDL (Table 3). This alteration was became significant 25 days after BDL. The time dependent alteration of rat liver weight and rat liver volume after BDL was shown in Figure 2. The difference between values of liver volume and wet liver weight was repeated in every volumetric analysis. The volumetric analyses indicated lower liver weights than measured wet liver weights. 

**Figure 3:**
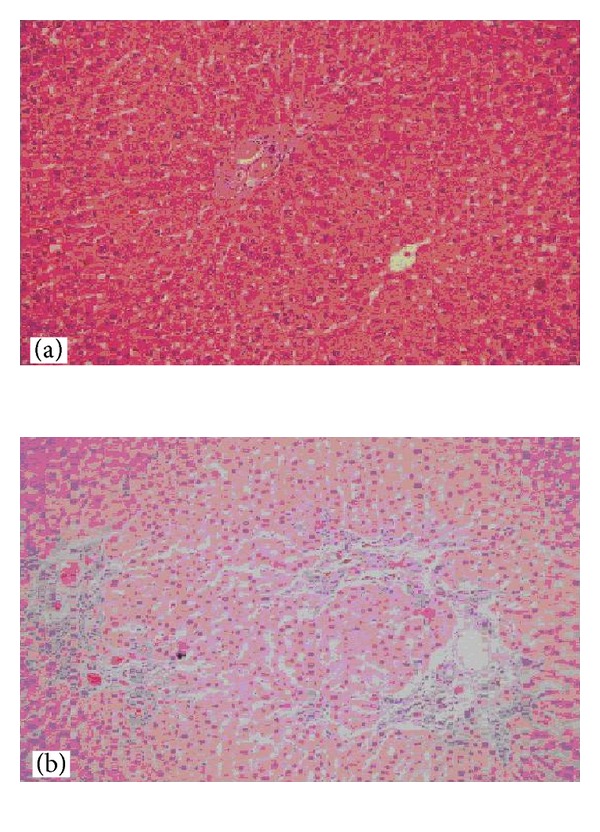
Examination under light microscopy with hematoxylin-eosin dye was demonstrated bile duct proliferation after BDL. (a) Preservation of lobular regularity was observed in control group (×200 HPF). (b) Prominent proliferation of bile ducts was observed in portal areas 25 days after BDL (×200 HPF).

**Figure 4:**
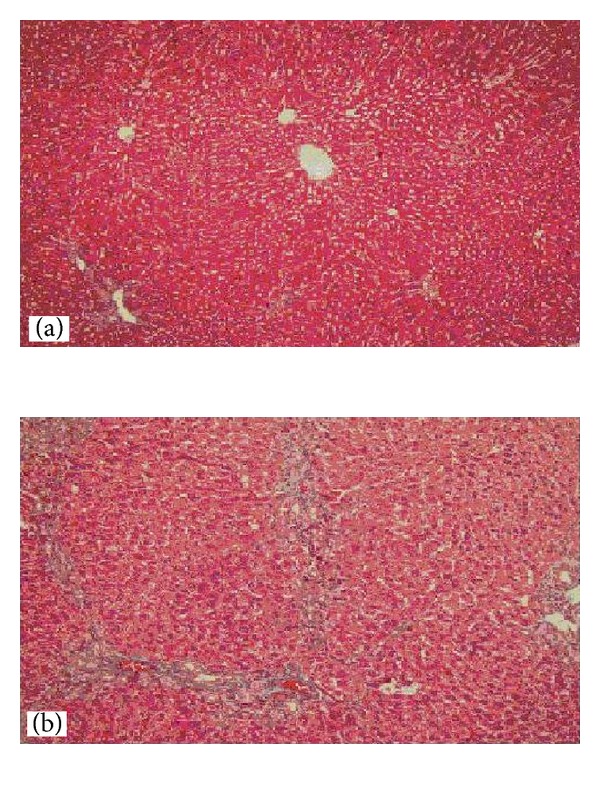
Examination under light microscopy with Masson's trichrome was demonstrated fibrotic alterations after BDL. (a) There was no fibrotic alteration observed in control group (×100 HPF). (b) Bridging fibrosis in the portal areas was observed 25 days after BDL (×200 HPF).

In general, proliferation of bile ducts and infiltration of inflammatory cells were commonly observed in the portal areas of all BDL rats. Inflammatory cell reaction increased in group 1 compared to other groups. The bile duct proliferation was the main cause of enlargement in portal areas 15 days after BDL (Figure 3). Hepatic fibrosis and bridging were identified 7 days after BDL. This finding was more evident 25 days after BDL (Figure 4).

## 4. Discussion

Numerous clinical and experimental studies have documented the presence of bile duct proliferation and hyperplasia after extrahepatic bile duct obstruction [1–9]. The onset of proliferation in bile duct epithelial cells is determined as early as 6 hours after BDL or biliary obstruction that results in a 60% increase in biliary ductal pressure [2]. In addition to the increased number of bile ducts, proliferating bile ducts are surrounded by spindle cells a couple of days after BDL [8]. The proliferating bile ducts tend to be infiltrating hepatic parenchyma within weeks after BDL. Hepatocytes in areas of proliferating bile ducts are atrophied within a month after BDL [8]. The increment of collagen types I and IV in hepatic extracellular matrix generally follows the proliferation process of bile ducts after BDL [3]. Whether collagen synthesis and deposition occur simultaneously with proliferation or as a response to it is also unknown. This collagen deposition is ended with irreversible fibrosis (cirrhosis) when the biliary obstruction is not relieved [4–6]. According to an experimental study by Zimmermann et al., dilated biliary ducts represent 11.6% of total liver parenchyma, and fibrosis represents 7.7% of total liver parenchyma after BDL [10]. Our clinical observations and results of the current study indicate enlargement of liver volume after BDL. Approximately liver volume and wet liver weight are increased up to 10% of original liver volume or weight 25 days after BDL. The origin of this liver remodeling is based on hepatic stellate cells (Ito cells, hepatic lipocytes, or fat-storing cells) [11, 12]. After liver injury, hepatic stellate cells undergo activation and transdifferentiation to myofibroblast-like cells. When activated by various cytokines, myofibroblast-like cells produce extracellular matrix [11, 13, 14]. We suggested that the abovementioned remodeling process of liver after BDL is reflected by enlargement of liver. 

The size of the liver is considered to be an important prognostic factor in patients with cirrhosis or fulminant hepatic failure, and imaging techniques have been used for obtaining quantitative measurements of liver volume [15, 16]. In patients scheduled for liver surgery for primary hepatic tumor, metastatic lesions, and transplantation, the liver volume must be known preoperatively [17–19]. The liver volume is one of the most important factors in the selection of appropriate donors, especially in a patient undergoing living-related liver transplantation (LRLT). Volumetry of the liver graft and remnant is mandatory for LRLT and is usually performed with cross-sectional computed tomography (CT) or magnetic resonance imaging. These methods yield reliable organ volume measurements when appropriate scanning protocols are used [19]. Volumetry of the liver on CT images is usually performed by manual tracing of the liver boundary and summation of the liver area on each section. Manual methods require considerable user involvement in the segmentation of the liver on each section, which is a time-consuming process. However, this approach is feasible when compared to the cost of fully automated hepatic volumetric analyses. Estimated volumetric data is generally expressed as cm^3^, and it is generally considered as equal to gr of wet liver weight [20, 21]. The accuracy of volumetric analyses is also very well studied in the literature. The strong correlation between volumetric analyses and wet liver weight is reported before [20]. In the current study, the CT scan machine produced for human analyses was used for volumetric study of rat liver. Therefore, this methodology was associated with limitation on accuracy of volumetric analyses. Although the manual tracing of the liver boundaries was performed by an experienced radiologist, the gap between wet liver weight and liver volume was almost constant during this study. All volumetric values were found lower than wet liver weight. In clinical studies, acceptable range of measurement error in volumetric analyses of the liver is not clearly described; however, it is generally considered as ±10% of actual liver weight in our routine clinical practice. The measurement error in this study was found to be more than 10% of actual liver weight. The gap was repeated constantly. Therefore, it is considered as a measurement bias by us. This difference may depend on the model of CT scan that is not prepared for animal studies. The radiologist, the designator of liver margins for volumetric analyses, was faced with some difficulties during measurement. The small size of rat liver, as clearly seen in Figure 2, hindered accurate estimation of liver volume. The manual tracing of liver boundaries was performed with a special software program for human studies. Also, we are not able to reduce the size of line used for drawing liver boundaries. The thickness of this line may cover too much area, and this factor may reduce the estimated liver volume than actual liver volume. Whether the cause of estimation gap depends on the CT scan unit or radiologist, the time dependent linear curve of volume increment after BDL was also observed. 

Various surgical procedures of the liver are performed with the adequate exposure of surgical anatomy of the liver. It is rational to control structures of portal hilus in addition to hepatic vein of involved lobe during hepatic resection. Extensive control of venous and arterial structures with total vascular exclusion is generally required in liver transplantation [22]. The enlargement of liver volume secondary to cholestatic liver injury causes exposure problems during liver surgery. In jaundiced patients requiring surgery for tumor resection, biliary drainage (BD) is suggested before hepatic resection for the elimination of negative effects of cholestasis in liver [23, 24]. The aim of preoperative BD is to improve liver function and reduce morbidity and mortality after radical surgery with major hepatectomy, that is, resection of more than three segments of the liver. The studies from Japan insisted that radical surgery be performed after complete recovery from jaundice, that is, a total bilirubin decrease to under 2.0 mg/dL [23]. It generally takes 4–6 weeks for liver function to recover after BD for jaundice [25, 26]. Despite the absence of published clinical data on alteration of overall liver volume after bile duct obstruction due to tumor, segmental or lobar atrophy is generally associated with type 3a or 3b hilar cholangiocarcinoma [27, 28]. The cause of this atrophy is generally related to infiltration of portal vein or hepatic artery of the ipsilateral liver lobe. However, Noie et al. demonstrate that bile duct ligation of the rat is associated with enlargement of the liver weight at the first week of BDL [29]. Authors also observed that liver weight returned to the preoperative level 1 week after complete biliary drainage. Selective application of BD caused segmental atrophy of nondrained segments of rat liver at 4 weeks of biliary drainage [29]. In patients with prolonged cholestatic injury, such as primary biliary cirrhosis, secondary biliary cirrhosis, or biliary atresia in infants, irreversible alterations occurre in liver parenchyma, and cirrhotic change is inevitable. 

In conclusion, our data indicate that BDL is inducing the increase of liver weight and liver volume. This effect is becoming prominent within the weeks after BDL. Proliferating bile ducts and fibrosis are the main determinants in the enlargement process of extracellular matrix.

## Figures and Tables

**Table 1:** Comparison of rat weights before and after experiment.

Groups	Before experiment (gr)	After experiment (gr)	*P* values
1 (*n* = 8)	239 (±24.6)	228.4 (±22.5)	0.13
2 (*n* = 8)	207.7 (±19.6)	207.6 (±30.4)	0.4
3 (*n* = 8)	222.6 (±9)	225.9 (±10.6)	0.3
4 (*n* = 8)	219 (±21.6)	234.4 (±22.8)	0.1

All values were expressed as mean ± SEM.

**Table 2:** Comparison of rat liver volumetric analyses before and after experiment.

Groups	Before experiment (cm^3^)	After experiment (cm^3^)	*P* values
1 (*n* = 8)	5.9 (±0.3)	6.1 (±0.3)	0.5
2 (*n* = 8)	4.5 (±0.3)	6.2 (±0.2)	0.01
3 (*n* = 8)	4.7 (±0.2)	7.2 (±0.5)	0.003
4 (*n* = 8)	5 (±0.2)	9.5 (±0.8)	0.002

All values were expressed as mean ± SEM.

**Table 3:** Comparison of wet liver weight in control group and experimental groups.

Groups	Wet liver weight (gr)	*P* values
Control (*n* = 4)	9.8 (±2)	
1 (*n* = 8)	10.4 (±0.3)	0.5
2 (*n* = 8)	11.4 (±0.6)	0.1
3 (*n* = 8)	11.9 (±0.9)	0.06
4 (*n* = 8)	14.1 (±1)	0.02

All values were expressed as mean ± SEM.

## References

[1] Morris JS, Gallo GA, Scheuer PJ, Sherlock S (1975). Percutaneous liver biopsy in patients with large bile duct obstruction. *Gastroenterology*.

[2] Slott PA, Liu MH, Tavoloni N (1990). Origin, pattern, and mechanism of bile duct proliferation following biliary obstruction in the rat. *Gastroenterology*.

[3] Tracy TF, Tector AJ, Goerke ME, Kitchen S, Lagunoff D (1993). Somatostatin analogue (octreotide) inhibits bile duct epithelial cell proliferation and fibrosis after extrahepatic biliary obstruction. *The American Journal of Pathology*.

[4] McPherson GAD, Benjamin IS, Hodgson HJF, Bowley NB, Allison DJ (1984). Pre-operative percutaneous transhepatic biliary drainage: the results of a controlled trial. *British Journal of Surgery*.

[5] Bhathal PS, Gall JAM (1990). Origin and involution of hyperplastic bile ductules following total biliary obstruction. *Liver*.

[6] Abdel-Aziz G, Rescan P-Y, Clement B (1991). Cellular sources of matrix proteins in experimentally induced cholestatic rat liver. *Journal of Pathology*.

[7] Beck PL, Lee SS (1995). Vitamin K1 improves survival in bile-duct-ligated rats with cirrhosis. *Journal of Hepatology*.

[8] Yoshioka K, Mori A, Taniguchi K, Mutoh K (2005). Cell proliferation activity of proliferating bile duct after bile duct ligation in rats. *Veterinary Pathology*.

[9] Aubé C, Moal F, Oberti F (2007). Diagnosis and measurement of liver fibrosis by MRI in bile duct ligated rats. *Digestive Diseases and Sciences*.

[10] Zimmermann H, Blaser H, Zimmermann A, Reichen J (1994). Effect of development on the functional and histological changes induced by bile-duct ligation in the rat. *Journal of Hepatology*.

[11] Gressner AM, Bachem MG (1995). Molecular mechanisms of liver fibrogenesis: a homage to the role of activated fat-storing cells. *Digestion*.

[12] Knittel T, Kobold D, Piscaglia F (1999). Localization of liver myofibroblasts and hepatic stellate cells in normal and diseased rat livers: distinct roles of (myo-)fibroblast subpopulations in hepatic tissue repair. *Histochemistry and Cell Biology*.

[13] Burt AD (1993). Cellular and molecular aspects of hepatic fibrosis. *Journal of Pathology*.

[14] Hautekeete ML, Geerts A (1997). The hepatic stellate (Ito) cell: its role in human liver disease. *Virchows Archiv*.

[15] Zoli M, Cordiani MR, Marchesini G (1991). Prognostic indicators in compensated cirrhosis. *The American Journal of Gastroenterology*.

[16] Sekiyama K, Yoshiba M, Inoue K, Sugata F (1994). Prognostic value of hepatic volumetry in fulminant hepatic failure. *Digestive Diseases and Sciences*.

[17] Okamoto E, Yamanaka N, Oriyama T, Tomoda F, Kyo A (1994). Prediction of the safe limits of hepatectomy by combined volumetric and functional measurements in patients with impaired hepatic function. *Cancer treatment and research*.

[18] Soyer P, Roche A, Elias D, Levesque M (1992). Hepatic metastases from colorectal cancer: influence of hepatic volumetric analysis on surgical decision making. *Radiology*.

[19] Kawasaki S, Makuuchi M, Matsunami H (1993). Preoperative measurement of segmental liver volume of donors for living related liver transplantation. *Hepatology*.

[20] Nakayama Y, Li Q, Katsuragawa S (2006). Automated hepatic volumetry for living related liver transplantation at multisection CT. *Radiology*.

[21] Kubota K, Makuuchi M, Kusaka K (1997). Measurement of liver volume and hepatic functional reserve as a guide to decision-making in resectional surgery for hepatic tumors. *Hepatology*.

[22] Birincioglu I, Topaloglu S, Turan N (2011). Detailed dissection of hepato-caval junction and suprarenal inferior vena cava. *Hepato-Gastroenterology*.

[23] Seyama Y, Makuuchi M (2007). Current surgical treatment for bile duct cancer. *World Journal of Gastroenterology*.

[24] Regimbeau JM, Fuks D, Le Treut Y-P (2011). Surgery for hilar cholangiocarcinoma: a multi-institutional update on practice and outcome by the AFC-HC study group. *Journal of Gastrointestinal Surgery*.

[25] Watanapa P (1996). Recovery patterns of liver function after complete and partial surgical biliary decompression. *The American Journal of Surgery*.

[26] Singh V, Kapoor VK, Saxena R, Kaushik SP (1998). Recovery of liver functions following surgical biliary decompression in obstructive jaundice. *Hepato-Gastroenterology*.

[27] McMaster PD, Rous P (1921). The biliary obstruction required to produce jaundice. *Journal of Experimental Medicine*.

[28] Hadjis NS, Adam A, Gibson R, Blenkharn JI, Benjamin IS, Blumgart LH (1989). Nonoperative approach to hilar cancer determined by the atrophy-hypertrophy complex. *The American Journal of Surgery*.

[29] Noie T, Sugawara Y, Imamura H, Takayama T, Makuuchi M (2001). Selective versus total drainage for biliary obstruction in the hepatic hilus: an experimental study. *Surgery*.

